# The Clinical Challenge of Autoimmune Psychosis: Learning from Anti-NMDA Receptor Autoantibodies

**DOI:** 10.3389/fpsyt.2017.00054

**Published:** 2017-04-19

**Authors:** Pierre Ellul, Laurent Groc, Ryad Tamouza, Marion Leboyer

**Affiliations:** ^1^DHU PePSY et Pôle de psychiatrie et d’addictologie des Hôpitaux Universitaires Henri Mondor, AP-HP, Université Paris Est Créteil (UPEC), Créteil, France; ^2^INSERM U 955, Equipe de Psychiatrie Translationnelle, Créteil, France; ^3^Fondation FondaMental, Fondation de coopération scientifique, Créteil, France; ^4^Institut interdisciplinaire de neurosciences, CNRS UMR 5297, Université de Bordeaux, Bordeaux, France

**Keywords:** schizophrenia, autoimmune diseases of the nervous system, microbiota, autoantibodies, treatment

## Introduction

Schizophrenia is a heterogeneous and complex psychiatric disorder affecting up to 1% of the population worldwide (1). Although the precise development of schizophrenia is not yet fully understood, it is now admitted to be underpinned by the entanglement of genetic, environmental, and immuno-inflammatory factors. Among schizophrenic patients, it is assumed that at least 30% will not respond to conventional antipsychotics (2). These data underlie the importance of precision medicine in psychiatry, in other words, the need to identify subgroups of patients with specific signatures who will benefit from treatment targeting these specific biological pathways. Reviving an area of exploration older than a century, recent and abundant literature emphasized the importance of the immune system in the pathophysiology of schizophrenia [for review, see Ref. (3)].

In psychiatry, the link between psychotic disorders, particularly schizophrenia, and immune system deregulations, including autoimmunity, is an old concept that regained strong support; thanks to the better characterization of brain inflammation-induced psychotic symptoms and autoimmune encephalitis (3). Moreover, recent epidemiological studies evidenced a high prevalence of multiple autoimmune diseases in schizophrenic patients (4). In a recent meta-analysis, autoantibodies against neuronal receptors have indeed been identified in the circulation of patients with neuropsychiatric disorders, constituting, today, one of the hottest topics in psychiatry (5–10). This new era fosters debate on (i) how to explain the increased burden of autoimmunity in schizophrenia, (ii) what could be the precise target(s) and the pathogenic implication(s) of the autoantibodies on the disease onset and development, (iii) how to define patient subgroups carrying such autoantibodies to facilitate their diagnosis, and (iv) how should we treat these patients using appropriate protocols such as immunotherapy (i.e., corticotherapy or plasmapheresis). Several neurological autoimmune diseases are, for instance, efficiently treated once autoantibodies against neurotransmitter receptors and ion channels have been identified (11, 12). The discovery of the autoimmune encephalitis due to anti-*N*-methyl-d-aspartic acid receptor (NMDAR) has greatly revived the relationship between autoimmunity and psychosis. Indeed, directed against the NMDAR *N*-methyl-d-aspartate receptors antibodies (NMDAR-Ab), the autoantibodies are directly responsible for the psychotic symptoms and catatonia, followed by profound neurologic deterioration (13, 14). In patients with schizophrenia, the prevalence and clinical significance of circulating NMDAR-Ab remains controversial with detection prevalence rates varying considerably between studies (15–27). Inspite of such imprecisions, defining and isolating seropositive patients, suffering from “autoimmune psychosis,” is a major challenge for appropriate treatments. In this review, we focus our attention on the potential elements possibly helping to define an “autoimmune psychosis” subgroup of schizophrenic patients. Furthermore, we outline some of the specific clinical presentation of these patients that will be of great importance to optimize the diagnostic and subsequent therapies.

## Autoimmunity and Psychosis: Roots

Autoimmune disorders occur after the failure of self-recognition processes with consequent production of pathogenic autoantibodies directed against specific or multiple organs. They are heterogeneous disorders, representing more than 80 different diseases. Several risk factors contribute to the high prevalence of autoimmunity including genetic and environmental ones and their interplay. Within this context, the “immunogenetic” contribution is largely dominated by the major histocompatibility complex (MHC) genetic diversity and, at a lesser extent, by mutational events affecting cytokines encoding genes (28). On the other side, environmental stressors are also of major importance for the onset of autoimmunity. For example, infections during pregnancy or in childhood are associated with an increased risk of type 1 diabetes (29). Moreover, and within the GxE context, it has been postulated that the risk of autoimmunity is enhanced through the perturbation of gut microbiota or dysbiosis (30, 31). This dysbiosis seems to be the origin of the emergence of different autoantibodies, even if the exact mechanisms involved are still under debate (32).

In psychosis settings, similar epidemiological associations with early infections, autoimmune disorders, and dysbiosis have pinpointed the possible existence of an autoimmune psychosis subgroup in schizophrenia. Maternal exposure to influenza or toxoplasmosis during pregnancy has been associated with schizophrenia. Childhood autoimmune diseases as well as inflammatory diseases, such as asthma, are known to be associated with an increased number of psychotic experiences in adolescence but also with an increased incidence of schizophrenia in the adulthood. Moreover, in patients with autoimmune conditions, the risk to develop schizophrenia increases linearly with the number of severe infectious episodes (4). The other way around, patients with schizophrenia and their first degree relatives, also exhibit a higher prevalence for autoimmune disorders (33). Last, associations between autoimmunity, gastrointestinal symptoms, and dysbiosis are starting to emerge (34). These data, along with the strong association between the interindividual immunogenetic background and the whole array of brain and peripheral autoantibodies, in at least a subgroup of schizophrenic patients, led us to propose the concept of “autoimmune psychosis.” Accordingly, our goal is to review the evocative characteristics that should prompt the search of autoantibodies in front of a patient, in particular, in cases of first episode, resistant ones, or schizophrenia with neurological comorbidity.

## Biological and Clinical Features of Patients with Autoimmune Psychosis

### Autoimmune Psychosis: Genetic and Environmental Risk Factors

Several genome-wide association studies (GWAS) confirmed an association between the MHC region (chromosome 6) and psychosis (35, 36). Moreover, a recent landmark GWAS analysis produced by the largest consortium on genetics of schizophrenia has shown that, like in autoimmune disorders, the MHC region was the most strongly associated (best *p*-value: MHC-region: *p* = 3.86e−32; 36,989 cases and 113,075 controls) (37, 38). The consequences of these mutations are still to be fully understood because some of them are found in non-coding region. However, in a matter of interest, the MHC region include the human leukocyte antigen (HLA) cluster, which is the most polymorphic and gene-dense genomic part of the human genome (39) encompassing more than 250 genes (4 Mb) and 14,000 alleles as reported to date (IMGT/HLA database; http://www.ebi.ac.uk/imgt/hla). Governing the specific adaptive immune responses, the HLA molecules were widely explored in disease-association studies (40) especially concerning those classified as autoimmune disorders (40–42). Even if more studies are needed to understand the link between immunogenetic and psychosis, disentangling such diversity might help to delineate the concept of autoimmune psychosis, at least on a genetic point of view.

On the other side, although data on gene–environment interactions are scarce, several environmental risk factors have been associated with schizophrenia and would be worth testing with MHC/HLA haplotypes. In particular, the occurrence of infections by pathogens such as, *influenza, herpes simplex type 2, cytomegalovirus*, and *Toxoplasma gondii* and/or increased C-reactive protein plasma levels during pregnancy are known to be associated with an increased risk of developing schizophrenia in adulthood (43–45). In the same context, hospitalization for infection increased the risk of schizophrenia by 60%, and there is a dose–response relationship between the number of hospital contact with infection and psychosis (46). Altogether, the reported deep intricacies between infection and autoimmunity, either under an additive or a more complex framework, with a consequent risk of psychosis, reinforce the concept of autoimmune psychosis concept (46).

More than separated risk factors, actual studies argue for a complex interaction between genetic risk factors conferring susceptibility to environmental injuries. For example, polymorphisms of the innate system genes, like IL1B, IL6, TNF alpha, or interferon, will lead to a bigger release of pro-inflammatory cytokines in response to environmental stressors (47).

In summary, immunogenetic dissection especially of the MCH/HLA region according to the natural history of deleterious immune processes including early infection and/or autoimmune features might be a promising route to better understand the interactions between gene and environment.

### Autoimmune Psychosis: Peripheral Biomarkers from Dysbiosis to Autoantibodies?

Similar to autoimmunity, dysbiosis is found in patients with psychosis (48). The intestinal microbiota seems essential for the development and functioning of the nervous central system, shedding light on the concept of a gut–brain axis (49). Dysbiosis is a well-known cause of increased intestinal permeability (so-called “leaky gut”) in schizophrenia (50). This increased intestinal permeability is demonstrated by the high circulating levels of CD14, a biomarker of bacterial translocation (49). The release of such pro-inflammatory innate sensor in a repetitive manner could allow, under the framework of particular genetic framework (HLA), to the breakdown of immune tolerance with consequent emergence of autoantibodies. Along this line, various autoantibodies have been found in subgroups of schizophrenic patients. For example, increased anti-bovine casein antibodies have been found in psychosis (51). Meta-analysis found threefold to fourfold times more anti-transglutaminase and anti-gliadine autoantibodies in patients with schizophrenia than in general population. Autoantibodies, specifically against the central nervous system, have also been found in schizophrenic patients. These patients have a higher prevalence of circulating antibodies against hippocampus and hypothalamus as compared to healthy control (52). A recent meta-analysis has confirmed and specified these results, showing that schizophrenic patients are three times more likely to have high levels of anti-glutamate receptor antibodies, *N*-methyl-d-aspartic acid receptor (NMDAR), compared to controls (22). The latter being of major importance. For the first time, they might make the bridge between autoimmune psychosis and the glutamate theory of psychosis and, doing so, sheds light on the pathophysiology of autoimmune psychosis.

In summary, there is a whole array of peripheral and central autoantibodies in schizophrenia, which deserve further exploration to explore their pathogenic role and to describe possible associated clinico-biological signatures, helping to more precise the concept of autoimmune psychosis.

### Autoimmune Psychosis: Clinical Picture?

We have seen that, among the heterogeneous group of schizophrenic patients, it is possible to hypothesize the existence of an autoimmune psychosis subgroup. The question is now in front of which clinical history or symptoms should we search for autoimmunity (53). The literature is still heterogeneous in the field and some have found no differences between patients (17, 20, 23). However, based on a French cohort of patients with psychiatric symptoms and autoantibodies against NMDA-R, we described clinical characteristics of patients that should lead to search of biological markers of an autoimmune psychosis [for details, see Ref. (54)]. While the mean age of onset in schizophrenia is 25–35 years old, we observed the first episode of autoimmune psychosis to occur around the 24th years of life (55). It is well known that schizophrenia is associated with the presence of neurological soft signs (56). More than that, we have been able to put forward that 50% of the patients with autoantibodies against NMDA-R had neurological symptoms including headaches, disorientation, paresthesia, anterograde amnesia, or abnormal movements. These results are in agreement with others who have also found neurological comorbidities in autoimmune psychosis cases (26, 57). Catatonia is a complex neuropsychiatric syndrome related to schizophrenia in 20% of cases (58). Its exact physiopathology is still unknown but seems underpinned by a deregulation between glutamatergic and GABAergic signaling (59). Catatonia, schizophrenia, and NMDAR-Ab have been extensively associated in the literature, which might indicate catatonia as a sign of autoimmune psychosis (60–62).

In summary, in front of an early age at onset of psychosis, discrete neurological symptoms, and catatonia, search for autoantibodies should be performed.

### Treatment Response

More than 30% of schizophrenic patients are resistant to conventional antipsychotics (63). Among them, 41% exhibited biological signs of immune activation (64). For example, treatment-resistant patient has been strongly associated with increased cytokines level (65–69). It has also been reported the specific presence of NMDAR-Ab in treatment-resistant patients (70). Finally, we have also been able to underlie the tight link between presence of NMDAR Ab and neuroleptic intolerance (54). These data seem to indicate a different pathophysiology, not related to the classical dopaminergic hypothesis, in patients with an autoimmune psychosis.

All these arguments have led to propose that all treatment-resistant/intolerant patients should have an autoantibodies screening, and particularly NMDAR-Ab, as a part of the diagnostic process.

**Figure 1:**
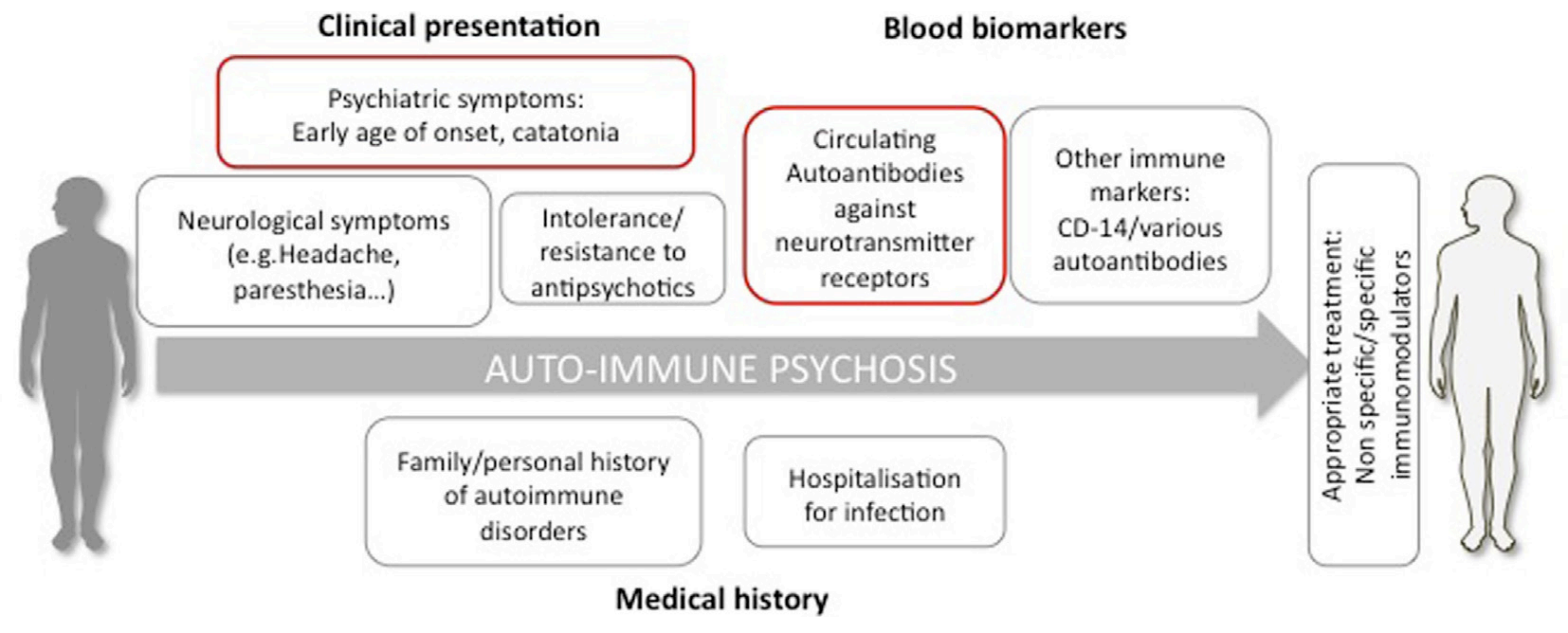
**Diagnostic elements possibly supporting the definition of autoimmune psychosis**. The diagnosis is suspected if the patient presents psychiatric symptoms (e.g., early age of onset, catatonia), neurological signs, resistant or intolerance to antipsychotic treatment, history of autoimmune disorder, and severe infections. The diagnosis relies on the detection in the circulation of autoantibodies and, particularly, directed against neurotransmitter receptors, such as the glutamate NMDA receptor.

In summary, based on epidemiological studies, genetic and biological biomarkers but also environmental risk factors, there are many arguments to suggest, that among schizophrenia, it is useful to ensure the identification of a subgroup of autoimmune psychosis. It is possibly characterized by (i) history of early infections or severe stress, (ii) autoimmune or infections during childhood or early adulthood, (iii) clinical presentation with the presence of gastrointestinal/neurological symptoms, catatonia, and (iv) presence of one or several autoantibodies associated with schizophrenia leading to a resistant form of schizophrenia (Figure 1).

## Perspectives for Appropriate Treatments

Now that we are able to isolate autoimmune psychosis, future clinical trials should evaluate if different types of immunotherapy may be helpful, in particular, involving those routinely used in immune/autoimmune-related common disorders such as cortisone pulse therapy, intravenous immunoglobulins, plasmapheresis, humanized monoclonal antibodies (e.g., rituximab), or immunosupressor (e.g., cyclophosphamide).

Among the new therapeutic approaches already used with success in autoimmune psychosis, three can be considered (10). The first one is based on the use of immunotherapies from non-selective immunosuppressive ones like minocycline, steroids, plasma exchange, or cyclophosphamide to a more selective one like the anti CD-20 monoclonal antibody Rituximab (71). Schematically, CD-20 is a potent marker of B-lymphocytes. By targeting CD-20, Rituximab will be able to inhibit B lymphocyte and, doing so, to prevent antibodies circulation (72). The second approach also focuses on antibodies and has been proposed by Diamond and colleagues. They propose to use d-peptide in order to prevent pathogenic antibodies to reach their target, theoretically, without affecting receptor function (73). It has been tested in mice model and seems to indeed prevent autoantibodies effect (73). The last one is more specific and consists in the use of a co-agonist of the NMDAR, the d-serine. It has been used by Heresco-Levy in an open label case study and has shown a dramatic improvement in the psychosis symptomatology (74). The potential mechanism of action behind d-serine is that it will enhance NMDAR activity by increasing the frequency of channel opening to counter act the action of the antibodies.

## Conclusion

Today, the main problem of the so-called autoimmune psychosis is that patients are not diagnosed. In order to help the physician to evocate it and to consider an autoantibody screening, we propose to gather elements enabling to build a risk score for autoimmune psychosis. This score should take into account the personal and/or familial history of early infections, autoimmune disorders, the demographic and clinical characteristics, and the presence of blood biomarkers such as CD14 and a panel of autoantibodies (anti-bovine casein, anti-transglutaminase, anti-folate receptor, anti-central nervous system, etc.). Of course, such risk score will need to be built and validated, as it should enable to allow early detection of autoimmune psychosis to prevent misdiagnosis with long-term deleterious consequences. Clinical trials targeting specific mechanisms and performed in homogeneous subgroups of autoimmune psychosis will allow to test and to select the most efficient treatment. More than that, the discovery of *N*-methyl-d-aspartate receptors antibodies (NMDAR-Ab) is also of major importance for a better comprehension of the neurobiological basis not only of autoimmune psychosis but also psychosis in general. We hope that, in a few years, personalized psychiatry will become the rule and not the exception anymore.

## Author Contributions

PE made the bibliography and wrote the article. ML, LG, and RT have corrected the manuscript.

## Conflict of Interest Statement

The authors declare that the research was conducted in the absence of any commercial or financial relationships that could be construed as a potential conflict of interest.
